# What’s new in protein kinase/phosphatase signalling in the control of plant immunity?

**DOI:** 10.1042/EBC20210088

**Published:** 2022-09-30

**Authors:** Jessica Erickson, Philipp Weckwerth, Tina Romeis, Justin Lee

**Affiliations:** Department for Biochemistry of Plant Interactions, Leibniz Institute of Plant Biochemistry, Weinberg 3, D-06120 Halle, Germany

**Keywords:** calcium-dependent protein kinases, kinase, mitogen-activated protein kinases, phosphatase, Phosphocode, signalling

## Abstract

Plant immunity is crucial to plant health but comes at an expense. For optimal plant growth, tight immune regulation is required to prevent unnecessary rechannelling of valuable resources. Pattern- and effector-triggered immunity (PTI/ETI) represent the two tiers of immunity initiated after sensing microbial patterns at the cell surface or pathogen effectors secreted into plant cells, respectively. Recent evidence of PTI-ETI cross-potentiation suggests a close interplay of signalling pathways and defense responses downstream of perception that is still poorly understood. This review will focus on controls on plant immunity through phosphorylation, a universal and key cellular regulatory mechanism. Rather than a complete overview, we highlight “what’s new in protein kinase/phosphatase signalling” in the immunity field. In addition to phosphoregulation of components in the pattern recognition receptor (PRR) complex, we will cover the actions of the major immunity-relevant intracellular protein kinases/phosphatases in the ‘signal relay’, namely calcium-regulated kinases (e.g. calcium-dependent protein kinases, CDPKs), mitogen-activated protein kinases (MAPKs), and various protein phosphatases. We discuss how these factors define a phosphocode that generates cellular decision-making ‘logic gates’, which contribute to signalling fidelity, amplitude, and duration. To underscore the importance of phosphorylation, we summarize strategies employed by pathogens to subvert plant immune phosphopathways. In view of recent game-changing discoveries of ETI-derived resistosomes organizing into calcium-permeable pores, we speculate on a possible calcium-regulated phosphocode as the mechanistic control of the PTI-ETI continuum.

## Introduction

**Figure 1:**
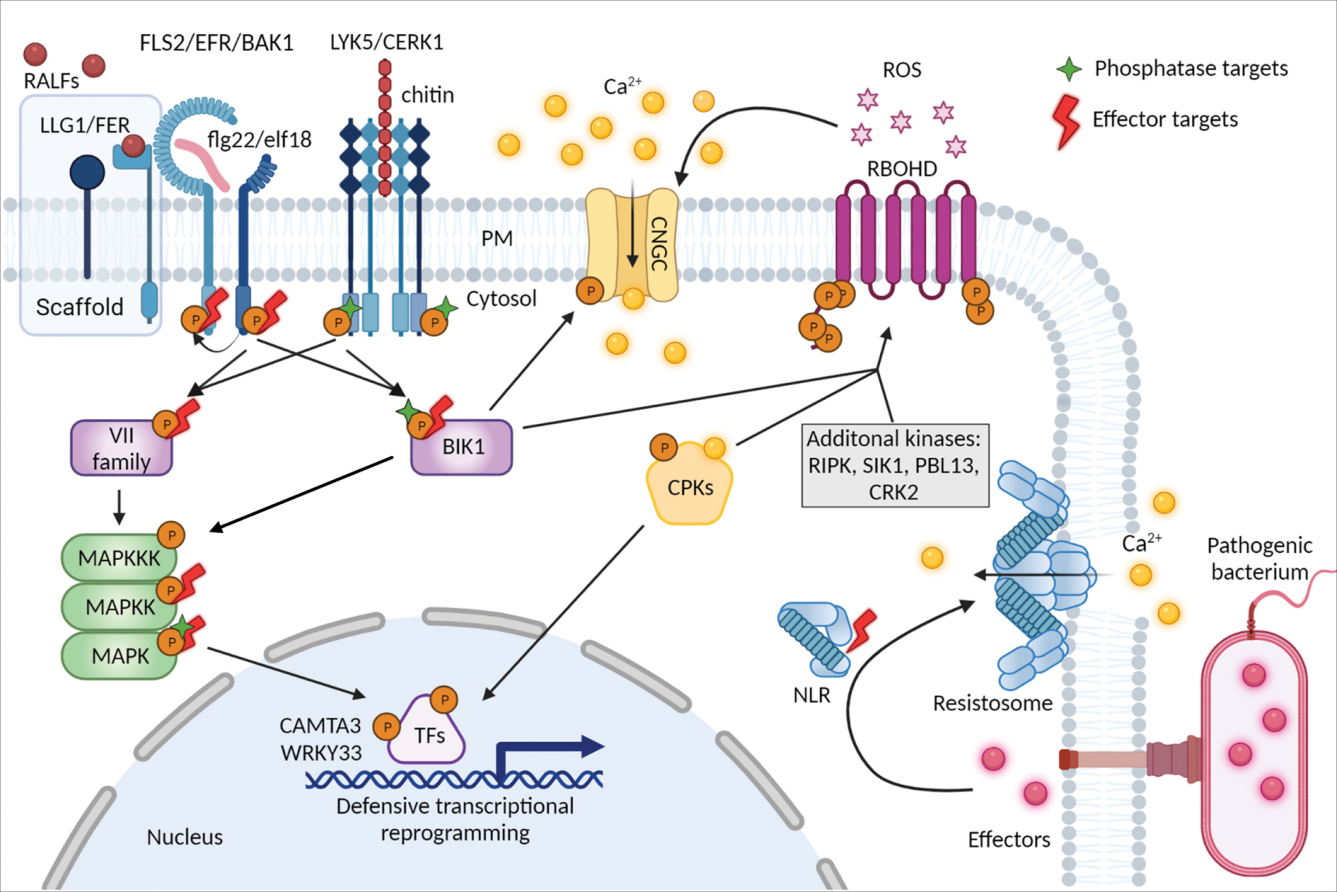
Overview of phospho-dependent immune signalling facilitated by endogenous protein kinases, edited by phosphatases, and manipulated by pathogen-derived effectors At the surface of the plant cell, pathogen-derived molecules (e.g. fungal chitin or bacterial flg22/elf18; in red) are recognized by PRR complexes comprised of RLK receptors LYK5, FLS2, or EFR (light blue) and coreceptors CERK1 and BAK1 (dark blue) embedded in the plasma membrane (PM). The FLS2-BAK1/EFR-BAK1 complexes are also regulated through membrane nanodomains assisted by the FER/LLG1 scaffold in a RALF-dependent manner. On the cytoplasmic side, phosphorylation (orange circles marked with P) activates PRR signalling, leading to the phosphorylation and release of RLCKs of the VII subfamily, including BIK1 (purple rectangles). Subfamily VII RLCKs activate the MAPK cascade (green), while BIK1 phosphorylation of CNGC calcium channels (yellow) and RBOHD (fuchsia,) activates calcium (yellow orbs) influx and ROS (fuchsia stars) production, respectively. CPKs (yellow pentagon) sense and decode the calcium signals and write the phosphocode on diverse targets. On the PM, CPK5, as well as additional protein kinases (gray box, and BIK1), mediate phosphorylation of RBOHD to guarantee ROS production, which can induce further calcium influx and thus form feed-forward calcium-ROS amplification loops. In the nucleus, CPK5 targets transcription factors, some of which are commonly phosphorylated by MAPKs (e.g. WRKY33 or CAMTA3). The distinct phosphosite specificities of MAPKs and CPKs generate a phosphocode-defined ‘logic gate’ that dictates transcriptional reprogramming during defense. Protein phosphatases act in opposition to protein kinases at multiple levels of the immune phosphocascade (targets shown as green stars), erasing the phosphorylation marks. Effectors (red orbs) injected into the cytosol by pathogenic bacteria may be recognized in resistant plants through NLRs (blue). Recently it was shown that an effector-modified RLCK serves as a ligand to trigger oligomerization of a coiled-coil-type NLR into a calcium-permeable pore, further increasing cytosolic calcium flux. In susceptible plants, effector activity mimics or hijacks endogenous control mechanisms to rewrite the phosphocode and ultimately suppress immunity at all levels (example targets mentioned in this review are marked with red lightning bolts).

In the ‘zig-zag’ model of plant immunity [1], the terms ‘**pattern-** and **effector-triggered immunity** (**PTI/ETI**)’ are used to designate immune systems initiated after recognition of pathogen-derived (or plant-derived) molecules at the cell surface or translocated effector proteins in the cytosol, respectively. PTI is mediated by cell surface pattern recognition receptors (PRRs) after sensing of conserved microbial molecules (so-called microbe-associated molecular patterns (MAMPs), e.g. EF-Tu, flagellin, peptidoglycans), or via the detection of endogenous plant molecules released from damaged tissues (damage-associated molecular patterns (DAMPs), e.g. AtPep1) [2]. Binding of these ligands to PRRs or PRR complexes belonging to receptor-like kinase (RLK) or receptor-like protein (RLP) families represents the first layer of immunity (Figure 1). To overcome PTI, many phytopathogens secrete/translocate effectors directly into the host cytosol, where they interfere with host immune pathways to support pathogen proliferation [3,4]. In resistant plants, effectors or effector activity is recognized via cytoplasmic nucleotide-binding leucine-rich repeat domain-containing receptors (NLRs), triggering a robust immune signalling cascade (ETI) that often culminates in local programmed cell death (the so-called hypersensitive response), presumably to limit pathogen spread [1]. Recent characterization of the coiled-coil-type NLR ZAR1 has shown that upon activation ZAR1 oligomerizes as part of the ‘resistosome’, a multicomponent resistance-triggering complex, which in this case forms calcium-permeable pores in the plant membranes [5,6]. ETI is normally of long-lasting and stronger intensity than PTI. However, PTI and ETI share several signalling components and are, in fact, more intertwined than previously thought. They may be viewed as a **defense continuum of increasing amplitude** rather than separate entities [7], but the mechanistic control of the PTI/ETI interplay is still unknown. Post-translational modifications (PTMs), such as defined phosphorylation pattern(s), maintained through the activity of specific protein kinases and phosphatases, could be a possible mode. Recent evidence of cross-potentiation between PTI and ETI [8,9] would support this notion. To bypass the constraints of the binary PTI/ETI definition, terms such as **surface-** or **intracellular-immunity** have emerged to designate the site of immune activation. In this spatially defined context, the plant immune system continuum can be extended beyond subcellular localization to **local/systemic immunity** at the plant level or even at the community level through interplant signalling mechanisms. Using this spatial definition, we summarize recent phosphorylation-related studies from the surface PRR complexes to the regulation of intracellular (de)phosphorylation and manipulation of these phosphopathways by pathogen effectors (graphically depicted in Figure 1). Phosphorylation generates a ‘protein mark’ at the modified site that encodes information representing different functional states of the protein. We discuss how such a **phosphocode**, particularly through multisite phosphorylation, could generate decision-making ‘logic gates’ and/or graded responses of tuneable amplitude. Finally, we highlight and speculate on how phosphoregulation of the calcium-ROS (reactive oxygen species) amplification loop may delineate signalling generated by the recently discovered calcium-permeable pores formed by ETI resistosome complexes in host membranes [5,6].

## Modulation of protein kinase recruitment and activity within PRR complexes initiates surface immune signalling

PTI signalling begins at the cell surface upon **PRR recognition of immunogenic microbial patterns**. Individual PRRs possess distinct ectodomains that bind ligands and convert this into intracellular signalling processes through phosphorylation events. To achieve this, RLPs, which lack a cytoplasmic kinase domain, would require protein kinase-containing partners for transmembrane phosphosignalling, while RLKs may directly employ their intracellular kinase domains. Yet, even RLKs show ligand-induced receptor/coreceptor oligomerization and recruitment/release of additional kinases (e.g. receptor-like cytoplasmic kinase (RLCK) family members) [10]. Some of the best studied receptor–coreceptor pairs include FLS2-BAK1, EFR-BAK1, LYK5/CERK1, and PEPR1-BAK1 (see glossary for full names), which detect MAMPs (flg22, elf18, fungal chitin) or DAMPs (AtPep1) [11]. On the cytoplasmic side, these PRR complexes is associated with the RLCK, Botrytis-induced kinase 1 (BIK1) [12], which is released from the complex after phosphorylation and monoubiquitination [13]. Reciprocal transphosphorylation has been shown for the receptor/coreceptor and BIK1. Importantly, phosphorylation in the activation loop of FLS2 or EFR plays a central role for full activation of downstream responses [14,15]. Most eukaryotic protein kinases are so-called RD-kinases, which harbor an arginine in the conserved catalytic loop of the HRD motif and require activation loop (auto)phosphorylation for full catalytic activity. FLS2 and EFR are, however, non-RD kinases as they lack this arginine and either do not need phosphorylation-dependent activation or employ other activation mechanism. Non-RD kinases typically have lower *in vitro* kinase activities compared with their RD counterparts [16]. Thus, the PRR **complex transphosphorylation** described above is consistent with the notion of the RD-kinase, BAK1, activating the non-RD kinases, FLS2 or EFR. Surprisingly, the catalytic activity of EFR cytoplasmic kinase domain was recently found to be dispensable for initiating antibacterial immunity although transphosphorylation of the EFR activation loop was essential for downstream signalling [17]. The authors proposed that it is phosphorylation-dependent **conformational changes** within EFR that initiate downstream signalling, for instance, by enhancing the BAK1 coreceptor activity. This challenges the above-mentioned concept of signal activation through activity enhancement of non-RD-type RLKs. It additionally questions the role of the kinase domain within the PRRs for the signal relay: can the substrates downstream of the PRR complexes be redundantly phosphorylated by either the receptor or coreceptor, or is this exception restricted to EFR?

Together with the above-mentioned EFR data, other recent studies are revealing that the regulation of kinase activities within PRR complexes is more complicated than previously thought. Feronia (FER), an RLK with malectin-like ectodomains, was found to regulate EFR-BAK1 or FLS2-BAK1 complexes independent of its intracellular kinase activity [18]. FER functions as a conventional receptor for sensing extracellular matrix or cell wall changes, where it forms (with its partner, Lorelei-like glycosylphosphatidylinositol (GPI)-anchored protein (LLG)) heterotypic PRR complexes to bind endogenous rapid alkalinization factor (RALF) peptides [19]. For immune regulation, FER ‘moonlights’ as a RALF-regulated **scaffold** to facilitate ligand-induced FLS2-BAK1 or EFR-BAK1 complex formation. Studies of single FLS2-GFP particle trajectories and their diffusion coefficients show that FER contributes to **membrane nanoscale spatial partitioning** and assembly of other PM PRR complexes [20]. Several additional examples of malectin-containing RLKs that regulate immunity by modulating immune PRRs have emerged recently (reviewed in [21]), so that the interplay between immune PRRs and these sensors that otherwise govern cell wall integrity, peptide hormone signalling, or other growth processes may contribute to growth-defense trade-off maintenance in plants. A future question will be to assess how the nanoscale partitioning of PRRs contributes to signalling fidelity of downstream phosphorylation events.

## Several intracellular protein kinases translate the surface immune signals into cellular phosphocode(s)

To transduce the danger signal from the surface PRRs to intracellular responses, two major cellular phosphorylation pathways are activated downstream of the PRR complexes, namely **mitogen-activated protein kinases** (MAPKs) [22] and **calcium-regulated kinases** [23,24]. In regard to the latter, emphasis will be placed on **calcium-dependent protein kinases** (CDPKs) [25]. Large gene families encode both MAPKs and CDPKs, with individual members contributing to signal specificities [22,25], and while both are protein kinases, they exhibit distinct activation mechanisms and target specificities. Enzymatic activity of CDPKs that are involved in immune signalling is directly enhanced after sensing calcium changes triggered by PRR activation [24] (see below). MAPKs operate as a multicomponent cascade, requiring phosphoactivation by an upstream MAPK kinase (MKK), which itself also requires another MKK kinase (MKKK) [26]. While an upstream MKKK kinase exists in some organisms and has also been proposed for plants [27], it has been unclear how MKKKs are activated after the immune PRR complex activation.

RLCKs, as central players in receptor complexes linking PRRs to downstream signalling, are obvious candidates to target MKKKs [28], but analyses have been complicated by functional redundancies among the huge numbers of RLCKs. For instance, little-to-no reduction in MAPK activation could be detected in double mutants lacking two related RLCKs, *BIK1* and *PBL1* [29], although these PRR-interacting RLCKs clearly contribute to PTI signalling. **PTI-relevant RLCKs** include the **family VII** (46 members) and family XII (12 members) [30]. Recently, systematic mutant analysis including higher-order mutants was undertaken [31–33] and clarified three previous uncertainties in the field: (I) Members of the RLCK-VII clade 4 indeed directly phosphorylate MKKK5 to positively regulate chitin-induced defense activation, thus finally revealing the **missing link between RLCKs and MKKK** activation. (II) Subsets of RLCK members are **differentially recruited** to distinct PRR complexes. EFR, FLS2, and BAK1 mainly engage BIK1 and PBL1 (clade 8) [10,29], whereas chitin signalling occurs through BIK1, several clade 4 members [15,31,32] and possibly PBL27 (clade 1) [33], while downstream signalling of lipooligosaccharide-specific reduced elicitation (LORE)-mediated detection of bacterial 3-OH fatty acid [34,35] occurs through PBL34-36 (Clade 5) [36]. (III) The MKKK upstream of the MKK4/5-MPK3/6 cascade is now identified to be MKKK3 and MKKK5 [31] rather than being assigned to MEKK1 [37]. Altogether, these findings show how distinct members of multigene families such as the RLCKs confer **signal specificity** by connecting the PRR complex to activation of distinct MAPK cascade components, thus tailoring the activation of different stages of the signalling cascade. Additionally, as described in the next section, RLCKs also trigger the release of additional signalling molecules such as calcium and ROS.

## Calcium and phosphorylation are tightly connected and feed into a calcium-ROS amplification loop

Downstream of PRR activation, elevation of cytosolic calcium levels and production of ROS are hallmarks of PTI/ETI signalling, with **calcium** and ROS production **closely linked to phosphorylation** [24,38]. A genetic screen with calcium as a read-out revealed BAK1 and membrane association of BIK1/PBL1 to be required for full response of MAMP-triggered calcium fluxes [29,39], thus hinting to (in)direct **phosphoregulation of the plasma-membrane-localized calcium channels**. Several cyclic nucleotide-gated channels (CNGCs) have been proposed to be the putative MAMP-responsive calcium channels; and indeed, CNGC2/4 were found to be direct substrates of BIK1 [40], while CNGC20 was phosphorylated by BAK1/SERK4 [41]. Similarly, the rice OsCNGC9 calcium-permeable channel, which is required for chitin-triggered calcium influx and resistance to the rice blast fungus, is phosphorylated by a rice RLCK-VII isoform RLCK185 [42]. Furthermore, channel activity of the calcium channel OSCA1.3 was also increased after BIK1-mediated phosphorylation in guard cells [43]. However, there is still uncertainty if any of these represent the genuine calcium channel(s) responding to MAMPs in Arabidopsis foliar tissues. The hunt for the elusive MAMP-responsive channel(s) operating under physiologically relevant conditions continues, but screening for channels targeted by BIK1/PBL1 or BAK1 may be a feasible strategy. Likewise, it is unknown if calcium signatures generated during ETI are mediated by the same channel(s) as PTI.

Various calcium sensors read and decode the PTI/ETI-induced calcium signature and directly or indirectly translate it into changes in phosphorylation capacity [23]. While other decoders such the tomato calcineurin b-like interacting protein kinase 6, CIPK6, translate calcium sensing into phosphorylation and play a role in plant immunity [44], we focus mainly on CDPKs here. CDPKs function as major calcium sensor-decoder in a single entity that directly transduce the MAMP-induced calcium signals into phosphorylation events. Like the other protein kinases mentioned above, CDPKs are encoded by a multigene family, with 34 members in Arabidopsis [25]. In Arabidopsis, the CDPK, CPK5, directly phosphorylates the NADPH oxidase RBOHD at distinct sites, which leads to an increase ROS production. Inversely, external ROS application leads to CPK5 activation and further RBOHD phosphorylation, thus forming a **calcium-phosphorylation-ROS amplification loop** [45]. Multiple protein kinases up-regulate RBOHD activity (see below). This includes BIK1, which is degraded after phosphorylation by another CDPK, CPK28 [46], thus attenuating RBOHD activation [47]. Hence, besides regulating plant growth [48], CPK28 prevents unwarranted immune activation. Interestingly, kinase-inactive CPK28 isoforms are generated by alternative splicing during flg22 activation of MPK4 [49] or DAMP signalling [50]. The resulting truncated CPK28 variant is thought to outcompete active CPK28, thus countering the negative regulation of BIK1 and enabling ROS generation [51].

RBOHD-derived ROS induces secondary calcium fluxes and contributes to the MAMP-induced calcium signature [39]. Altogether, calcium fluxes and ROS accumulation are interlinked with several intracellular protein kinases (RLCKs, CDPKs, and MAPKs, and additional kinases mentioned below) for maintaining and amplifying immune activation. In particular, the CPK5-mediated immune signal can propagate systemically to induce systemic acquired resistance (SAR) in distal tissues [45] and is accompanied by accumulation of the SAR-inducing metabolite N-hydroxy-L-pipecolic acid (NHP) [52]. The physical association of CPK5 with the truncated NLR, TN2 (TIR-NBS2), also pinpoints CPK5 to be a vital signalling node for ETI [53]. Hence, protein kinases from PTI signalling and the calcium-ROS amplification loop may determine the **phosphocodes for PTI, ETI, and** possibly even **SAR**.

## A regulatory phosphocode through multisite phosphorylation

**Figure 2:**
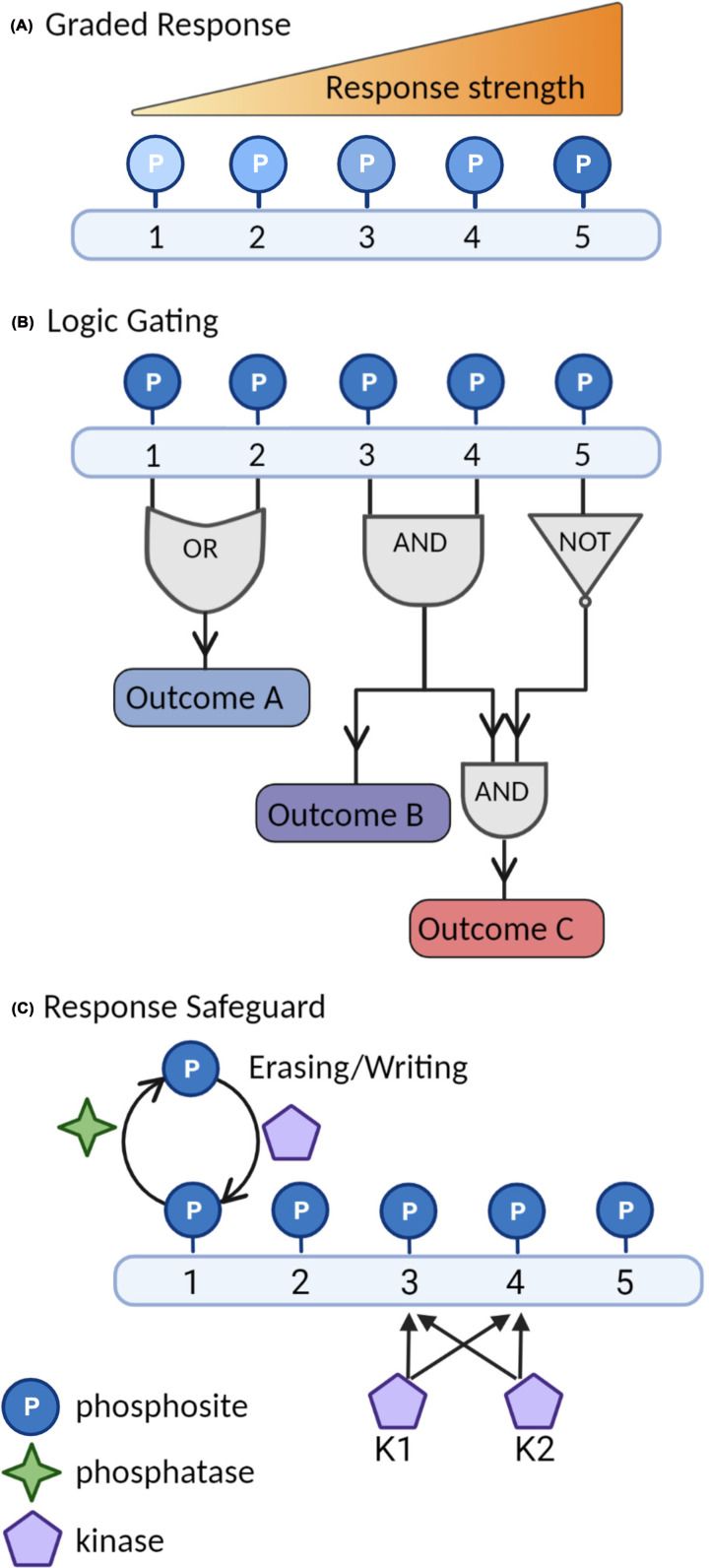
Regulatory phosphocode through multisite phosphorylation Schematic models of how multisite phosphorylation on a single protein (conceptually valid for both protein kinases and their substrates) constitutes a regulatory phosphocode. (**A**) Sequential or consecutive phosphorylation by one or different protein kinases may generate a **graded response** that is tuneable by actions of opposing protein kinases and phosphatases present. An example is when all the phosphomarks result in the same outcome such as degradation (exemplified by MAPK substrates mentioned in this review), where the frequency of modified phosphodegron motifs will correlate with the likelihood of engagement by ubiquitin-proteasome machineries, and therefore increased removal as outcome. (**B**) **Logic gating** represents more complex decision-making that is important for phosphocode-dependent regulation of protein functions. Three hypothetical scenarios are illustrated here: (1) Outcome A arising if either phosphosite 1 OR 2 are modified; (2) Outcome B occurs only if both phosphosite 3 AND 4 are phosphorylated; or (3) Outcome C, a follow-up situation where, additionally, site 5 must NOT be phosphorylated. Phosphocode-dependent subfunctionalization of BAK1 or CPK28 or the convergence of MPK3/6 and CPK5 on transcription factors WRKY33 and CAMTA3 represent such situations. (**C**) **Response safeguard** represents the scenario where multiple independent protein kinases target one or more key phosphosite(s) required for full activity of the substrate protein. Using Outcome B (in b) as an example, if K1 is inactivated, a safeguarding second protein kinase (K2) with overlapping phosphosite specificity will ensure activity maintenance. An example for this scenario is the convergence of several protein kinases on RBOHD.

While most studies have focused on the impact of individual phosphosites on protein functions, phosphoproteomics revealed many proteins to be multiphosphorylated [54]. What is the cumulative outcome of multisite phosphorylation compared with the ‘on/off’ signal transduced by single-site phospho-switches? A current view is that multisite phosphorylation can provide a **graded and tuneable response** that is dependent on the level of the input protein kinase and its opposing phosphatase (Figure 2A). If protein kinases and phosphatases from different biological pathways and contexts are involved, this further generates a cellular decision-making ‘**logic gate**’ (Figure 2B). Increasingly complex logic gating occurs if **antagonistic cross-talk** exists between phosphosites where one phosphosite inhibits or promotes phosphorylation of other sites in the same network [55]. Hence, each phosphosite forms a protein mark that constitutes a phosphocode for information processing and this principle applies to several of the immunity-relevant phosphoproteins. Notably, aside from the substrate proteins, the protein kinases or phosphatases themselves are often subjected to phosphocode-dependent regulation—sometimes through auto-(de)phosphorylation. In its simplest form, multiphosphorylation of a distinct target protein can be mediated by a single protein kinase **sequentially**, which could theoretically form a temporally defined phosphocode [56]. Several substrates of flg22-responsive MAPKs are phosphorylated at multiple sites by a single MAPK, where the phosphosites contribute to protein stability [57–60]. This represents a biochemical conundrum of multiple site accessibility for the protein kinase, and raises the question of whether substrates dislodge and re-engage for consecutive phosphorylation. While still not thoroughly investigated, one may draw analogy to sequential multisite phosphorylation by yeast cyclin-dependent kinase 1 (Cdk1), which, like MAPKs, is a proline-directed protein kinase. For Cdk1, multiphosphorylation is propagated in an N-to-C-terminal direction along a disordered region of the substrate [61]. In the above-mentioned plant examples, variants mutated in all MAPK-targeted sites were more stable than ‘partial’ mutants of some sites [57,58,60]. If each phosphosite represents a phosphodegron motif, multisite modification may increase the rate of proteasome engagement and represents one means by which a ‘**graded response**’ is achieved.

When several protein kinases converge on a single substrate, the corresponding multisite phosphocode develops into a powerful signal processing ‘**logic gate**’ circuitry (Figure 2B). This becomes relevant for components shared by different signalling pathways, such as BAK1 and CPK28, where the distinct phosphosite(s) relevant for immune functions or growth regulation were recently identified [62,63]. Thus, signalling fidelity of BAK1 and CPK28 can be explained by this **phosphocode-dependent subfunctionalization**. A similar control mechanism may be assigned to other immune signalling hub proteins, such as RIN4, which is phosphorylated by several kinases and additionally, post-translationally modified by ADP-ribosylation and acetylation; for a recent review, see [64]. An excellent example for multisite phosphorylation is RBOHD, where its activity is positively regulated by multiple (flg22-responsive) protein kinases, including CPK5 [45], BIK1/PBL1 [65,66], RIPK [67], SIK1 (an MKKK kinase) [27], and cysteine-rich RLK2 (CRK2) [68]. Here, it is noteworthy that several protein kinases converge on S343 and S347, possibly acting as a **manifold safeguard mechanism** (Figure 2C) to ensure high RBOHD activity. By contrast, the RLCK, PBL13, targets T912 of RBOHD to promote proteasome-dependent degradation [69], thus functioning as a negative regulator of ROS production. Likewise, the two major flg22-responsive cellular protein kinase pathways, CDPK and MAPK, also converge on at least two immune relevant transcription factors [57,70]. Since these protein kinases have distinct phosphosite specificities, the resulting phosphocode can define the ‘logic gate’ decision. Indeed, DNA binding and transactivation activities of WRKY33 are separately promoted through phosphorylation by CPK5/6 and MPK3/6, respectively [70], so that these kinases co-operatively control the WRKY33-regulated biosynthesis of camalexin to inhibit microbial growth. For CAMTA3, MPK3/6 regulates its destabilization and nuclear export during PTI [57], while the effect of CPK5 has not been fully elucidated. However, the role of CPK5 in NLR signalling [53] could mean that the convergence of MAPKs and CDPKs on CAMTA3 constitutes a phosphocode-defined rheostat for the PTI-ETI continuum. Supporting this notion is the identification of CAMTA3-binding sites in overlapping PTI/ETI defense genes that imply CAMTA3 defines an early convergence point in NLR- and PRR-signalling [71].

The evolution of multiple protein kinases to modify common targets underscores the importance of such signalling nodes, which as discussed above, can contribute to **signalling fidelity, strength, and duration**. By contrast, phosphorylation can also provide interpathway cross-talk/interplay. An example being immune priming between different pathogen pathways, such as flg22 treatment inducing enhanced antifungal immunity or antiviral activity through phosphorylation-dependent stabilization of their corresponding receptors, CERK1 [72] and NIK1 [73]. Taken together, multikinase-mediated phosphocode enables complex decision-making in signalling.

## Rewriting the immune phosphocode through protein phosphatases as erasers

While protein kinases write the phosphorylation marks, counteracting phosphatases serve as **erasers to change the defense phosphocode**, which is essential for preventing unnecessary or excessive immune signalling. While the broad-spectrum activity of many plant protein phosphatases and functional redundancies have hindered the definition of their specific roles, there is increasing evidence of stringent regulation by specific protein phosphatases at multiple levels of the immunity phosphoproteome. Among the 150 annotated Arabidopsis protein phosphatases, most studies have focussed on Ser/Thr phosphatases or dual-specific (Ser/Thr and Tyr) phosphatases and much less is known about protein Tyr phosphatases [74]. Starting with the PRR complexes, kinase-associated protein phosphatase (KAPP) associates with and attenuates signalling of the wall-associated kinase 1 (WAK1) receptor for DAMPs; the *kapp* mutants show correspondingly increased resistance against *Botrytis cinerea* [75]. Similarly, PRR co-receptors are also negatively regulated by phosphatases, e.g. BAK1 through PP2A holoenzyme [76] or CERK1^Y428^ dephosphorylation by CERK1-interacting protein phosphatase 1 (CIPP1) [77]. The phosphatase PP2C38 was initially identified as an interactor of EFR and FLS2 but, rather than inactivating the receptors, it modulated the phosphorylation and activation status of BIK1, thereby preventing immune signalling prior to elicitation [78]. Interestingly, PP2C38^S77^ (feedback) phosphorylation by its own substrate, BIK1, is needed for BIK1-PP2C38 dissociation from the PRR complex. Recent data also pinpoint similar mechanism for additional PP2Cs, namely poltergeist-like 4 and 5 (PLL4 & 5) where PLL4 dissociation is preceded by phosphorylation through BIK1 [79].

Similar feedback control is also observed for the protein phosphatases that inactivate the intracellular protein kinases such as MAPKs. In a feed-forward loop, stability of the MAPK phosphatase 1 (MKP1) is promoted by MPK6-mediated phosphorylation, thus promoting MPK6 dephosphorylation and shutting down of defense responses [80]. While the dual-specificity MKP1 dephosphorylates both ser/thr and tyr, inactivation of MAPKs through ser/thr phosphatases such as the protein phosphatase 2C (PP2C), AP2C1 [81] or various members from the type-one protein phosphatase (TOPP) family has also been shown to compromise plant resistance [82], although it cannot be excluded that additional non-MAPK targets may contribute to the disease phenotype. Nevertheless, the importance of these phosphatases for immunity is indirectly implicated in genetic screens, such as that identifying MKP1 to be required for *Plectosphaerella cucumerina* fungal resistance [83] or the absence of TOPP4 triggering an ETI-like cell death phenotype [84].

Currently, there are fewer examples of protein phosphatases that target the key immunity-related calcium-regulated protein kinases. Although not previously associated with immunity, CPK1 activity was dampened after dephosphorylation by the protein phosphatase, PP2A-B’γ, thereby affecting resistance to *Botrytis cinerea* [85]. Comparable to RBOHD, the paralogous RBOHF is activated by multiple protein kinases, including open stomata 1 (OST1) and two calcium-regulated protein kinases, CIPK11 and CIPK26, which is counteracted through dephosphorylation by the phosphatase ABI1 (ABA-insensitive 1). Immunity through stomatal closure may thus be regulated by the RBOHF-mediated ROS generation [86]. Here, it is noteworthy that the ABI family of PP2Cs seem to have additional roles other than being simply ABA coreceptors. ABI1, as well as additional phosphatases, ABI2 and HAB1, reversed the MAPK and/or CDPK-mediated activation of the rate-limiting enzyme for ethylene biosynthesis, 1-aminocyclopropane-1-carboxylate synthases, ACS6 [87] and ACS7 [88]. Similarly, ABI2 antagonizes the calcium-regulated CIPK5 phosphorylation of the guard cell outward rectifying k^+^channel (GORK) that is involved in jasmonic acid (JA)-induced stomatal closure [89]. Hence, the ABI PP2Cs may intersect into many other signalling pathways than currently assumed. Collectively, erasing phosphorylated protein marks by protein phosphatases may mediate (hormonal) signalling interplay and buffer the phosphocode for tight immunity control.

## Hijacking and manipulation of immune phosphocode by pathogen effectors

The balance of multiple protein kinases and phosphatases acting on signalling nodes facilitates the tight regulation of immune-pertinent phosphocascades to ensure activation at appropriate time points and amplitudes. Among the mechanisms that pathogens use to counteract plant defense, several converge on and hijack phosphoregulatory mechanisms to suppress host immunity. Over the last 20 years, the intense study of phytopathogen effector proteins has revealed more than a handful of effectors capable of directly altering the phosphocode via intrinsic kinase, phosphatase, or lyase activity [36,90–92]. Additionally, a vast array of effectors employ alternative enzymatic strategies, such as uridylation [93], ubiquitination [94], and ADP-ribosylation [95] to indirectly manipulate phosphorylation. Since surface immunity is the first line of inducible plant defense [1], it is unsurprising that successful pathogens harbor multiple effector proteins that suppress one or more of these early phospho-dependent PTI components (reviewed in [3]; Figure 1). Among the 28 effectors injected by *Pseudomonas syringae* strain DC3000 (*Pto*), at least 5 (HopF2; [95], HopB1; [96], AvrPto; [97], AvrPtoB; [94,98], HopAO1; [36,99]) directly **interfere with phosphorylation** of PRR complex components at the membrane and several effectors (HopF2; [95], HopAI1; [92], AvrRpt2 [100]) block downstream MKK or MAPK activities. For instance, HopAO1 (a tyrosine phosphatase) was shown to dephosphorylate EFR at Y836 [99] and, more recently, the PRR LORE at Y600, the phosphorylation of which is critical for the activation of downstream RLCKs PBL34/35/36 [36]. Such effectors act to erase the phosphocode of the immune component, a modification that is irreversible in some cases, e.g. the phosphothreonine lyase HopAI1 that dehyroxylates the T residue of MAPKs and prevents rephosphorylation needed for activation [92].

Besides interfering with phosphorylation, **mimicry of plant protein kinases** represents a novel strategy for manipulating the defense phosphoproteome to compromise plant immunity. For example, XopC2 (*Xoc*) and HopBF1 (*Pto*) were recently identified as representatives of two new clades of ‘atypical’ protein kinases. The XopC2 family harbors additional α-helix subdomains making the kinase domain atypically long (470 aa vs. the typical 265 aa; [91]), while HopBF1 homologs exhibit a minimal protein kinase domain (only 183 aa; [101]). In an elegant study, Wang et al. (2021) revealed that XopC2 directly phosphorylates the SCF complex adaptor protein, OSK1 (at Ser53), resulting in the activation of jasmonate signalling and suppression of stomatal immunity [91]. HopBF1 (*Pto*), on the other hand, inhibits ETI through the phosphorylation and resultant inactivation of the chaperone protein Hsp90. The authors suggest that Hsp90 phosphorylation ultimately prevents proper folding of client proteins such as NLRs and/or kinases important for signalling [101].

In addition to mimicking and inhibiting protein kinase activity to suppress immunity, some effectors hijack plant phosphopathways by **mimicking substrates** of conserved protein kinases to facilitate their own phosphorylation/activation. For example, in susceptible plant species, the phosphorylation of *Pto* effectors, AvrPto (S149) and HopQ1 (S51), via unidentified plant kinases, and subsequent recruitment of the plant phosphoform-recognizing 14-3-3 proteins to HopQ1, contribute significantly to their virulence functions [102–104]. Recently, AvrPtoB was found to interact with several plant protein kinases, including CDPKs as well as members of sucrose nonfermenting 1 (SNF1)-related kinases (SnRKs), SnRK 1.1, 2.6, and 2.8. In this study, SnRK 2.8 was found to be the primary player for AvrPto phosphorylation at S258, S210, and S258, which proved essential to its virulence function [105].

The evolution of multiple effectors, employing a variety of strategies, to converge on PRRs, MAPK elements and other signalling nodes only serves to emphasize their importance. However, given that calcium influx and decoding of this signal by CDPKs are equally crucial to both PTI and ETI [106,107], it is surprising that detailed studies of effectors that directly target the calcium-sensing or calcium-regulated phosphorylation, e.g. through CDPKs or CIPKs have, thus far, not been frequently reported. Although some putative CDPK-effector interactions have been mentioned in the literature (e.g. between truncated versions of CPK4/5 with *Pto* effector AvrPtoB [105] or CPK6 with three *Xanthomonas campestris* pv. *campestris* effectors, XopK, XopAC, and XopJ [108]), these have not been followed up. In view of the recent revelation that several NLRs form calcium-permeable channels [5,6], we propose that effectors that **target elements of calcium signalling** deserve more attention in the future.

## Conclusion and future perspectives

In this review, we highlighted recent advances in our molecular understanding of phosphorylation-dependent immune regulation. We also highlight the need to further explore interplay/cross-talk between complex phosphocoding (e.g. between phosphosites of multiphosphorylated proteins) and additional PTMs - either through plant machineries or pathogen effectors. A current proteomics bottleneck for this endeavor is the analytic limitation to obtain full coverage of all PTM events in a given protein at a specific time, especially for low-abundance proteins. Besides technical advances, a holistic approach combining structural biology (cryoelectron microscopy, cross-linking/MS, and nuclear magnetic resonance), machine-learning-based phosphosite prediction tools and structural modelling (e.g. AlphaFold) will be beneficial to decipher the PTI/ETI phosphocode. For instance, how does ETI trigger a stronger and more sustained calcium influx and phosphosignalling (e.g. MAPK activation profile) compared with PTI? The demonstration of shared signalling components and interdependence between PTI and ETI [8,9,109] further endorses the notion of immunity as a PTI-ETI continuum.

Three recent breakthrough discoveries in the understanding of ETI components will likely pave the way for future understanding of how calcium fluxes and perception shape immunity. First is the previously mentioned resistosome, which is made up of coiled-coil-type NLR pentamers that form calcium-permeable pores in host membranes [5,6]. Second is the finding that for the second class of Toll-interleukin-1-receptor (TIR)-domain NLRs, immune activation triggers tetramerization of the TIR domain to form a NAD-hydrolyzing enzyme that generates, among others, cyclic ADP-ribose (cADPR)-like molecules [110–113]. cADPRs are well-known calcium-releasing agonists. Third is that the so-called helper NLRs that function downstream of the TIR-type NLRs also oligomerize into calcium-permeable pores [114]. Thus, one can anticipate that these NLR-mediated calcium fluxes will feed into calcium-phosphorylation-ROS amplification loops described above: a likely explanation for the strong and sustained intensity of ETI responses. However, how does the cell decode a generic signal such as calcium without erroneous cross-talk into other calcium-regulated pathways (e.g. abiotic stress)? Here, a calcium-regulated phosphocode probably determines the signalling specificity and subsequent fine-tuning. Do the differential calcium sensitivities of decoders such as CDPKs [52], which are in part regulated by autophosphorylation [115], play a role in the decoding? Are there mechanisms to close/inactivate these pores to redefine an ETI-specific calcium signature and/or to prevent runaway cell-death through uncontrolled calcium toxicity? Finally, does the calcium signalling generated by the NLR pores lead to the sustained MAPK activation typically seen for ETI and if so, are (and which) RLCKs involved to phosphorylate the MKKKs? These and additional questions have been posed recently [116] and await future clarifications from the plant immunity community.

## Summary

- Intracellular protein kinases/phosphatases control immunity in the PTI-ETI-SAR continuum.
- Multisite phosphorylation (of substrate proteins but, often, also their corresponding protein kinases and phosphatases) defines the phosphocode for downstream mechanistic interpretation of immune signalling.
- Phosphocodes enable tuneable ‘graded response’ or more complex ‘logic gating’ to control response outcomes
- In light of recently discovered calcium influx generated by resistosome pores or calcium-mobilizing molecules, understanding the phosphocode edited through calcium-regulated enzymes will be central in future studies to dissect immune signalling.
- Effectors manipulate multiple points of immune phosphosignalling but those targeting calcium-sensing processes deserve greater attention.

## Abbreviations

ABI: ABA-insensitive
ACS: 1-Aminocyclopropane-1-carboxylate synthase
AP2C1: Probable protein phosphatase 2C 1
AtPep1: *Arabidopsis thaliana* Peptide 1
AvrPto: Avirulence Pto-interacting
BAK1: Brassinosteroid-associated kinase 1
BIK1: Botrytis-induced kinase 1
cADPR: cyclic ADP-ribose
CAMTA3: Calmodulin-binding transcription factor 3
Cdk1: cyclin-dependent kinase 1
CDPK: calcium-dependent protein kinase
CERK1: Chitin elicitor receptor kinase 1
CIPK: CBL-interacting protein kinase
CIPK6: Calcineurin b-like interacting protein kinase 6
CIPP1: CERK1-interacting protein phosphatase 1
CNGC: Cyclic nucleotide-gated channel
CPK: Calcium-dependent protein kinase
CRK2: Cysteine-rich RLK 2
DAMP: damage-associated molecular pattern
EF-Tu: elongation factor Tu
EFR: Elongation factor-Tu receptor
elf18: elongation factor Tu peptide
ETI: effector triggered immunity
FER: Feronia
flg22: flagellin 22 peptide
FLS2: Flagellin sensing 2
GORK: Guard cell outward rectifying K^+^ channel
HAB1: Hypersensitive to ABA 1
Hop: Hypersensitive response and pathogenicity outer protein
Hsp90: Heat shock protein 90
JA: jasmonic acid
KAPP: Kinase-associated protein phosphatase
LLG1: Lorelei-like glycosylphosphatidylinositol anchored protein 1
LORE: Lipooligosaccharide-specific reduced elicitation
LYK5: LYSM-containing receptor-like kinase 5
MAMP: microbial-associated molecular pattern
MAPK/MPK: mitogen-activated protein kinase
MAPKK/MKK: MAPK kinase
MAPKKK/MKKK: MKK kinase kinase
MEKK1: MAPK/ERK kinase kinase 1
MKP1: MAPK phosphatase 1
MS: mass spectrometry
NHP: N-hydroxy-L-pipecolic acid
NIK1: NSP-interaction kinase 1
NLR: nucleotide-binding, leucine-rich repeat receptors
OSCA1.3: Reduced hyperosmolality-induced calcium increase 1.3
OSK1: *Oryza sativa* SKP1-like
OST1: Open stomata 1
PBL: PBS1 (AVRPPHB susceptible)-like
PLL4/5: Poltergeist-like 4 or 5
PP2A: Serine/threonine protein phosphatase 2A
PP2A-B’λ: Serine/threonine protein phosphatase 2A 59kDa regulatory subunit B’gamma isoform
PP2C38: Protein phosphatase 2C 38
PRR: pattern recognition receptor
PTI: pattern triggered immunity
PTM: post-translational modification
*P*to: *Pseudomonas syringae*
RALF: Rapid alkalinization factor
RBOHD: Respiratory burst oxidase homologue D
RBOHF: Respiratory burst oxidase homologue F
RIPK: RPM1-induced protein kinase
RLCK: receptor-like cytoplasmic kinase
RLK: receptor-like kinase
RLP: receptor-like proteins
ROS: reactive oxygen species
SAR: systemic acquired resistance
SCF: Skp, Cullin, F-box containing complex
SERK4: Somatic embryogenesis receptor-like kinase 4
SIK1: Serine/threonine kinase 1
SNRK: Sucrose non-fermenting 1 (SNF)-related kinase
TIR: Toll-interleukin-1-receptor
TN2: TIR-NBS (nucleotide-binding site) 2
TOPP4: Type-one serine/threonine protein phosphatase 4
WAK1: Wall-associated protein phosphatase 1
WRKY33: WRKY DNA-binding protein 33
Xop: Xanthomonas outer protein
